# Blower-Powered Soft Inflatable Joints for Physical Human-Robot Interaction

**DOI:** 10.3389/frobt.2021.720683

**Published:** 2021-08-24

**Authors:** Ryuma Niiyama, Young ah Seong, Yoshihiro Kawahara, Yasuo Kuniyoshi

**Affiliations:** ^1^ Graduate School of Information Science and Technology, The University of Tokyo, Tokyo, Japan; ^2^ Graduate School of Engineering, The University of Tokyo, Tokyo, Japan

**Keywords:** inflatable robot, physical human-robot interaction, soft robotics, soft mechanism, tendon wire

## Abstract

Inflatables are safe and lightweight structures even at the human scale. Inflatable robots are expected to be applied to physical human-robot interaction (pHRI). Although active joint mechanisms are essential for developing inflatable robots, the existing mechanisms are complex in structure and it is difficult to integrate actuators, which diminish the advantages of inflatables. This study proposes blower-powered soft inflatable joints that are easy to fabricate and contain enough space for an actuation inside. The joints are driven by tendon wires pulled by linear actuators. We derived a theoretical model for both unilateral and bilateral joints and demonstrated a hugging robot with multiple joints as an application of the proposed joint mechanism. The novelty of the proposed joint mechanism and the inflatable robot is that rigid parts have been thoroughly eliminated and the tendons for actuation have been successfully hidden inside. Moreover, the active control of the internal pressure makes inflatables resistant to punctures. We expect that the contact safety of inflatable robots will facilitate advancement of the pHRI field.

## 1 Introduction

Inflatable structures are used in a variety of products such as rubber boats, mattresses, advertising columns, and temporary architectures. Their lightweight, cost-effectiveness, safety, and portability enhance the potential of inflatable robots. However, the major challenge with their application is the active kinetic mechanism, which is the difference between existing inflatable products and inflatable robots.

An early application of inflatable structures in robots was an attempt to replace the links of a space robot arm with inflatable cylinders (Koren and Weinstein, 1991). In this case, the joints and grippers were rigid mechanical parts. Studies were also conducted on an inflatable link manipulator with pneumatic bag actuators around the joints (Kim H.-J. et al., 2018; Liang et al., 2018). In a study on a long, lightweight inflatable arm filled with helium gas (Takeichi et al., 2017), thin McKibben pneumatic artificial muscles were used to drive the joints, again along the surface of film balloons. Otherlab Inc. built several inflatable robots using a combination of fabrics and air bags, and sewed fabric-based pneumatic actuators, called Peano actuators, to the inflatables to cause flexure (Sanan et al., 2014). Pneubotics and Otherlab Inc. also used a pair of pneumatic bladders for flexion and extension actions in a single-axis joint and humanoid robot (Best et al., 2015). For fabric-based inflatables, a spherical robotic arm that combines two rotational degrees of freedom (DoF) in a single joint with a triplet actuator has been reported (Hofer and D’Andrea, 2020).

To bend an inflatable, a large moment is required to induce buckling. A special structure is necessary to make an inflatable joint without any constrictions or separations. Air-Octor, a continuum robotic arm, had a pressured flexible duct as its backbone, and the three-dimensional bending was achieved by pulling the surrounding wires (McMahan et al., 2005). The joint mechanism with a constant volume, similar to the design of a spacesuit, has a complex bellows structure that allows the diameter of the joint to remain unchanged and has no partition walls (Voisembert et al., 2013).

Contact safety is important in considering the application of inflatable robots. Sanan *et al.* proposed a manipulator with both links and joints made of inflatable structures and focused on the physical interaction with humans (Sanan et al., 2011). The joint part of this manipulator was a sealed inflatable, and the wires for the actuation had to be placed outside the inflatable. Sensors for inflatable robots have also been developed to detect contact interactions (Kim T. et al., 2018). In the movie “Big hero 6”, the inflatable robot Baymax was a healthcare robot. In the context of human-computer interaction (HCI), an inflatable robot with built-in touch sensors and a projector is being developed in collaboration with therapists as a learning and play companion for children (Garzotto et al., 2017). An overview of related works shows that a few joint mechanisms for inflatable robots have been proposed, although their structures are complex, and it is difficult to enclose the actuators inside. Moreover, a simple joint mechanism driven from the inside is needed to realize an inflatable robot with multiple joints that can safely interact with humans. Another major concern with inflatable robots is air leakage due to small holes or cuts and the resulting malfunction. In the case where the support structure of the robot consists only of inflatables, the structure cannot maintain its strength when the internal pressure lowers. One solution is a hybrid structure, i.e., a separate skeletal structure, with the inflatable as the exterior (Ohta et al., 2017; Alspach et al., 2018). However, the hybrid structure cannot collapse like a bare inflatable unless it has a telescopic skeletal structure.

In this regard, to create an inflatable that is robust against air leaks, we focused on inflatables that are constantly pressurized by an electric blower. Most of the inflatable robots used pressurized and sealed bags, resulting in punctures as well as difficulties in accessing the inside of the inflatable. In contrast, blower-inflated balloons are puncture-resistant because they do not require a strict sealing and can be opened and closed for actuation and wiring. As an advantage, it is also possible to actively change the internal pressure of the inflatable by controlling the blower.

Some inflatable robots have been reported in patent databases. For example, a patent on an inflatable robot arm for a mobile robot was filed by iRobot Corporation (Annan et al., 2016). Another patented inflatable kinetic structure is the undulating figures known as the tube man (Gazit and Dranger, 2001). In the 1980s, the Japanese artist Shiro Takahashi created large kinetic sculptures using blower-inflated balloons. He registered a patent for a basic articulation mechanism for inflatable sculptures and now expired (Takahashi, 1987). Our preliminary technical report (Seong et al., 2019) presents the first quantitative evaluation of the mechanism proposed by Takahashi. In the report, the inflatable joints were limited to two sizes and were unilateral. In addition, the theoretical model was incomplete.

This paper presents comprehensive experimental results on groups of newly developed unilateral and bilateral inflatable joints of various sizes and ranges of motion. The inflatables were pressurized by an electric blower, with feedback control of the internal pressure. We also proposed an improved theoretical model and compared its predictions with the measured results. The advantages of inflatables are safety, lightweight, and portability by folding. As an application, we targeted physical HRI and demonstrated an example of a contact experiment between a human and a humanoid robot.

## 2 Blower-Powered Inflatable Joints

### 2.1 Basic Structure

**FIGURE 1:**
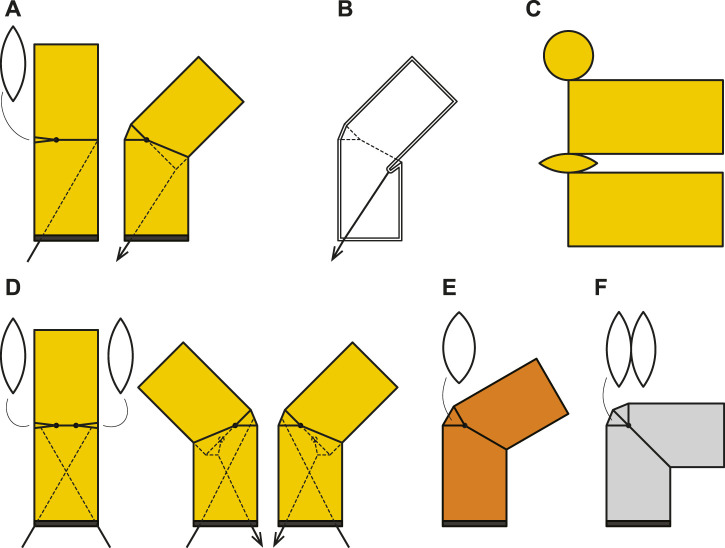
Forms and structures of inflatable joints. **(A)**: an unilateral joint with a single leaf-shaped patches and a tendon shown in side view, **(B)**: Internal structure and the pocket formed when the tendon is pulled, **(C)**: Pattern of unilateral joint for sewing. **(D)**: a bilateral joint with two leaf-shaped patches and two tendons, **(E)**: an unilateral joint with wide a patch, and **(F)**: an unilateral joint with a doubled leaf-shaped patch.

**FIGURE 2:**
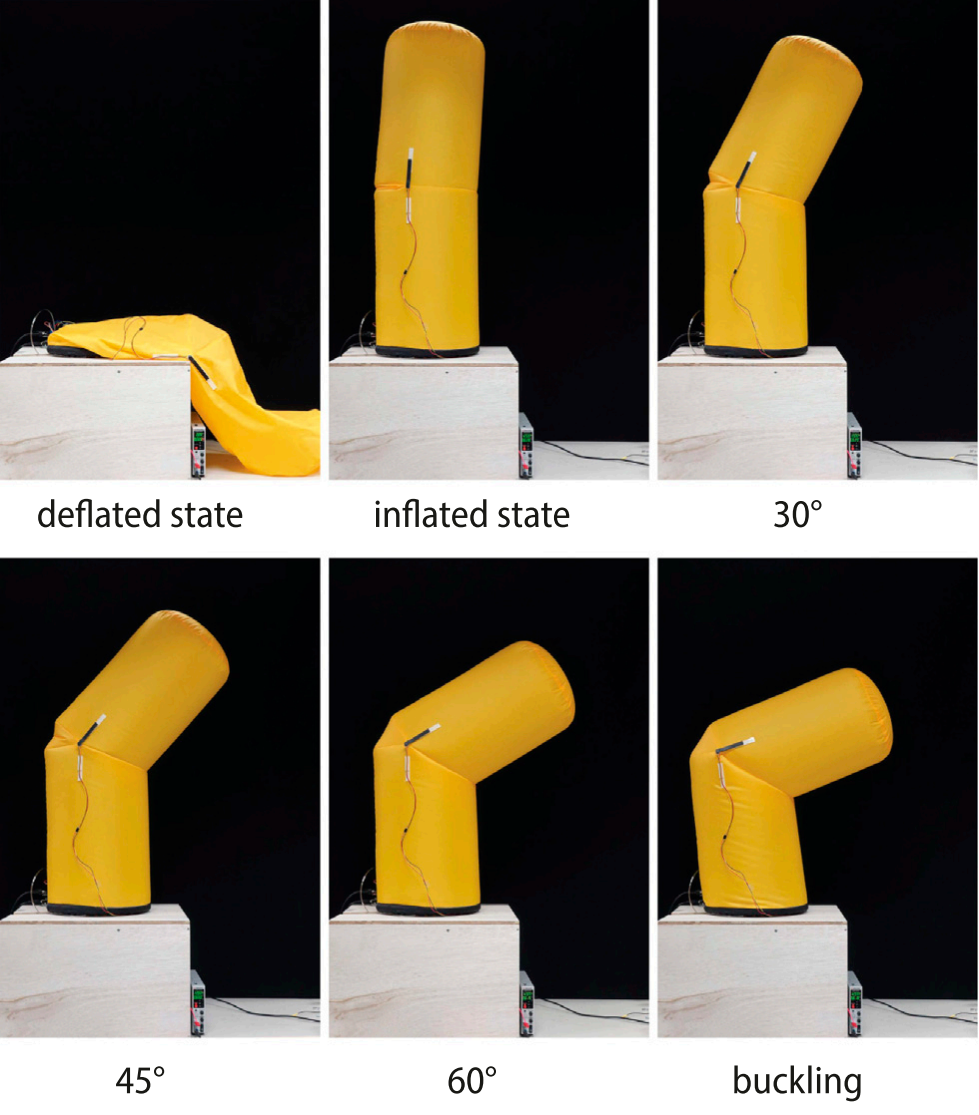
Motion sequence of an inflatable joint: deflated, inflated, flexion, and buckling.

We focused on the articulation mechanism installed in the middle of the cylindrical inflatable structure (Figure 1). The essential element of the proposed joint was to insert a leaf-shaped patch in the middle of the cylindrical inflatable to provide an additional surface area for bending. Unilateral joints have one leaf-shaped patch and can only bend on one side from the initial state (Figure 2A). Bilateral joints have leaf-shaped patches on both sides that can bend to either side from the initial state (Figure 2D). The size of the leaf-shaped patch is related to the range of motion (Figures 2E,F).

The joint bends when pulled by a tendon wire from the inside, and when the tendon is pulled, the wall is retracted to create a pocket, while a leaf-shaped patch unfolds on the other side (Figure 2B). We employed a low-pressure inflatable that was constantly pressurized by a blower, and some air leakages were acceptable. The tendons that drive the joints could be pulled out of the inflatable through small holes.

The unique feature of the proposed mechanism is that the joint section is made of the exact same material as the body of the inflatable. The diameter of the joint is the same as that of the body, and there are no internal partitions. We assume that the material for the inflatable could be a plastic film or fabric that is lightweight and flexible and equally has low elongation and minimal air leakage.

### 2.2 Theoretical Model of the Inflatable Joints

We propose a quasi-static model of a soft inflatable joint, which always operates in equilibrium and is not affected by the inertia of the membranes or the airflow dynamics. The model considers idealized membranes and ignores the thickness, mass, folding energy, and friction between membranes.

**FIGURE 3:**
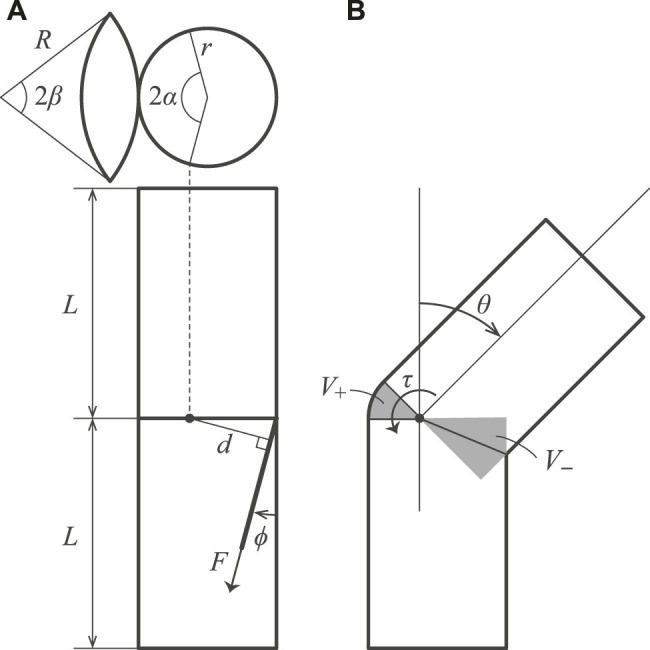
Geometric model of the inflatable joint. Lateral views of neutral and bending states, and a leaf-shaped patch are shown.

Figure 3 shows the design parameters of the inflatable joint. If the volume of the joint does not change during flexion, no energy is required. On the other hand, the recovery force *τ* is generated when the volume of the joint at an internal pressure *P* changes with flexion. We shall denote the volume of the inflatable joint by *V*(*θ*). The law of conservation of energy leads to the following equation.

PdV=−τdθ (1)

The left-hand side of the equation denotes the work done by the air with pressure *P* for an infinitesimal change in the volume d*V*. The right-hand side expresses the work done by torque *τ* around the pivot of the joint for an infinitesimal change in the angle d*θ*.

The torque *τ* can be derived as the follows:

$$\tau \left(\theta \right)=-P\frac{dV}{d\theta }$$ (2)

We let *V*_+_ and *V*_−_ be the volumes that increase and decrease due to bending, respectively. We assume that the leaf-shaped patch is spherical under internal pressure and that the radius of the body is represented by *r*. The following equations are used for calculating the volume:

$$\begin{array}{ll} V & =V_{+}-V_{-} \end{array}$$ (3)

$$\begin{array}{ll} V_{+} & =r^{3}\theta \left(\sin\alpha -\frac{1}{3}\sin^{3}\alpha -\alpha \cos\alpha \right) \end{array}$$ (4)

$$\begin{array}{l} \begin{array}{cc} V_{-} & =2r^{3}\tan\left(\frac{\theta }{2}\right)\left\{\frac{2}{3}\sin^{3}\alpha +\cos\alpha \left(\cos\alpha \sin\alpha +\pi -\alpha \right)\right\} \end{array} \end{array}$$ (5)

From the Eqs 2, 3 the torque that tries to return the joint to a neutral position is given as follows:

$$\begin{array}{ll} \tau & =r^{3}P\left(A\frac{1}{\cos^{2}\left(\frac{\theta }{2}\right)}-B\right) \end{array}$$ (6)

$$\begin{array}{ll} A & =\frac{2}{3}\sin^{3}\alpha +\cos\alpha \left(\cos\alpha \sin\alpha +\pi -\alpha \right) \end{array}$$ (7)

$$\begin{array}{ll} B & =\sin\alpha -\frac{1}{3}\sin^{3}\alpha -\alpha \cos\alpha \end{array}$$ (8)

Here, *A* and *B* are constants. The derived model indicates that the properties of the joint are not related to the length of the body. The *τ* obtained above can be applied to both unilateral and bilateral joints.

The relationship between the torque *τ* and the tension of the tendon *F* can be expressed as follows.

τ=Fd (9)

Here, *d* is the moment arm, *ϕ* is the angle of the tendon, and *α* is a variable that determines the offset of the rotation axis. For the moment arm, unilateral case *d*_*u*_ and bilateral case *d*_*b*_ need to be considered separately. This is because, in unilateral joints, the tendon pulls directly on the wall, while in bilateral joints, the tendon is attached to the center of a leaf-shaped patch that is folded inward. Assuming that the offsets due to the patch are *r*_*patch*_ and *θ*_*patch*_, the moment arm can be calculated as follows.

$$\begin{array}{ll} d_{u} & =r\cos\left(\left|\phi -\theta \right|\right)\left(1+\cos\alpha \right) \end{array}$$ (10)

$$\begin{array}{ll} d_{b} & =\left(r-r_{patch}\right)\cos\left(\left|\phi -\left(\theta +\theta_{patch}\right)\right|\right)\left(1+\cos\alpha \right) \end{array}$$ (11)

From Eqs 6, 9, 10, 11 the tension required to drive the joint is given as follows:

$$\begin{array}{ll} F_{u} & =\frac{\tau }{d_{u}} \end{array}$$ (12)

$$\begin{array}{ll} F_{b} & =\frac{\tau }{d_{b}} \end{array}$$ (13)

The volume *V* decreases monotonically with flexion under condition *α* ≤ *π*/2. The torque *τ* acts to return the *θ* to zero, and the joint behaves like a torsion spring. The torque *τ* increases as the offset of the rotation axis from the center of the inflatable increases. The parameter *α* sets the offset of the rotation axis. For a special case where *α* = *π*/2, Eqs 7, 8 are rewritten as follows:

$$A^{\prime }=B^{\prime }=\frac{2}{3}$$ (14)

In this case, the torque *τ* is relatively small compared to a joint with a small *α*. In other words, the joint bends with less force, and the effect of returning to a neutral posture is reduced.

For the geometry of the leaf-shaped patch, the following constraints need to be satisfied.

rα=Rβ (15)

Herein, the radius of the arc that forms the leaf-shaped patch is denoted as *R* and the central angle as *β*. Since the width of the patch defines the range of motion of the joint, the combination of *R* and *β* should be chosen appropriately. This issue is discussed in detail in the section on experiments with patches of different widths.

## 3 Experiments

### 3.1 Inflatable Samples

We prepared inflatables using various parameters for the experiments. Details of the parameters for samples of different sizes and different rated ranges of motion are shown in Table 1. Each inflatable has a single joint in the middle of the cylindrical body. The unilateral inflatable joint has one leaf-shaped patch, which is folded in a neutral state and unfolded in a flexion state. The bilateral inflatable joints have two leaf-shaped patches, and the joint can be bent to both sides from the neutral state. Tendon cables were attached inside the joint and we could control the inflatable joint by pulling this tendon. The inflatables were fabricated by sewing several nylon woven fabric parts together with a sewing machine based on the pattern shown in Figure 2C. The weight of the fabric is about 70 g/m^2^. The stitches were joined by straight stitches, and no leak-proof seal was applied, although a small amount of air may leak naturally. A fabric that is slightly thicker than the fabric that constitutes up the body is sewn on the inside and tied to the tendon wire.

**TABLE 1:** Details of inflatable samples.

Label	type	*r*	*R*	*α*	*β*	*L*	rated range
U200	unilateral	100 mm	2*r*	2/5*π* (= 72°)	1/5*π* (= 36°)	300 mm	45°
U300	unilateral	150 mm	2*r*	2/5*π*	1/5*π*	450 mm	45°
U300W	unilateral	150 mm	1.5*r*	2/5*π*	4/15*π* (= 48°)	450 mm	60°
U300D	unilateral	150 mm	2*r*	2/5*π*	1/5*π*	450 mm	90°
U300C	unilateral	150 mm	2*r*	1/2*π* (= 90°)	1/4*π* (= 45°)	450 mm	45°
B300	bilateral	150 mm	2*r*	2/5*π*	1/5*π*	450 mm	±45°
U400	unilateral	200 mm	2*r*	2/5*π*	1/5*π*	600 mm	45°

Regarding the scaling limitations, we have already tested prototypes ranging from a minimum height of 100 mm and a diameter of 70 mm to a maximum length of 3 m and a diameter of 0.5 m. Further study is needed for larger sizes, but scaling up and down is basically influenced by the strength and pliability of the fabric, the size and output power of the actuators, and the size and flow rate of the blower.

The internal pressure of the inflatable was set to less than 300 Pa. An inflatable structure made from lightweight fabric needed about 50–100 Pa to stand. The higher the internal pressure, the larger the moment the inflatable could tolerate. At high pressure, the risk of tearing from the seams occured.

A small value of the parameter *α* increased the force required for flexion. We chose a value of *α* close to 90°, which provided an practical extension torque. The parameter *R* was related to the height of the leaf-shaped patch and determines the rated maximum angle of the joint. The joint could be flexed beyond the rated angle, which distorted the body. We set the *R* such that the desired rated angle could be obtained and the pattern could be simple. Once the parameters *α* and *R* are determined, the *β* can be determined from Eq. 15.

### 3.2 Method

We measured the tensile force of the tendon, the travel distance, and the angle of the joint during flexion and extension while controlling the internal pressure of the inflatable joint. The measurement cycle was repeated, starting from the neutral position, pulling the tendon until near the rated range of motion or close to buckling, and then returning to the neutral position again. The experiment was performed under quasi-static conditions: the tendon pull speed was limited to 5 mm/s. The results were plotted for ten consecutive steady-state cycles, excluding the first cycle. All signals were low-pass filtered at a cutoff frequency of 0.5 Hz.

**FIGURE 4:**
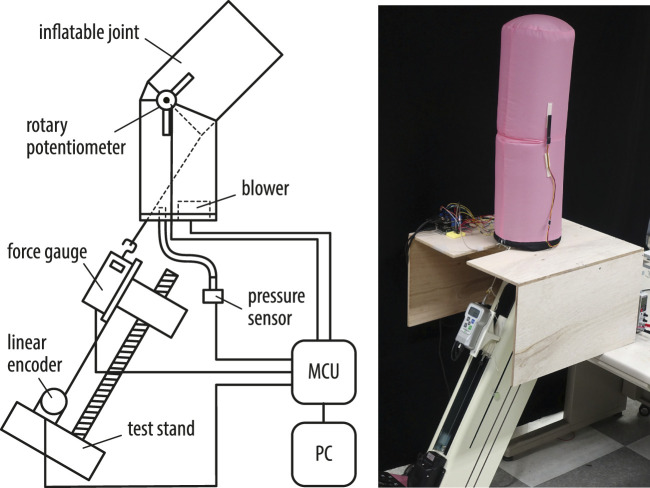
Schematic diagram of the experimental setup and a photo of the measurement.

Figure 4 shows the experimental setup. The system consists of a force gauge (FGP series, Nidec-Shimpo Inc.), an electric force test stand (FGS-250VC, Nidec-Shimpo Inc.), a wire encoder (D-1000Z, Mutoh Industries Ltd.), a pressure sensor with the range from 0 to 40 mbar (HSC series, Honeywell International Inc.), and a blower (San Ace B97 series, Sanyo Denki Inc.). A small protractor incorporating a rotary potentiometer (SV01 series, Murata Manufacturing Co., Ltd.) is attached to the surface of the inflatable with double-sided tape at the sharp corner of the leaf-shaped patch that serves as the axis of rotation. A micro-controller unit (Arduino Mega 2560 R3) collects measurements at a sampling frequency of 4Hz and sends them to the computer. The electric test stand can control the position of the force gauge connected to the tendon. The pulling angle is adjusted according to the experiment, and the force gauge moves back and forth at a constant angle. The tendon was long enough such that changes of a few degrees in the pulling angle during flexion could be ignored.

The pressure sensor measures the internal pressure of the inflatable through a tube. The blower is attached to the bottom plate and is inside the inflatable. We used blowers with a PWM control function. The MCU performs feedback control to keep the internal pressure constant based on the experimental conditions. The control cycle was 20 Hz. A blower with a maximum pressure of 1,280 Pa (9BMB12P2K01) was used for the 200 and 300 mm diameter inflatables, and a blower with a maximum pressure of 1950 Pa (9BMC12P2G001) was used for the 400 mm diameter inflatable.

### 3.3 Results

#### 3.3.1 Size and Pressure

**FIGURE 5:**
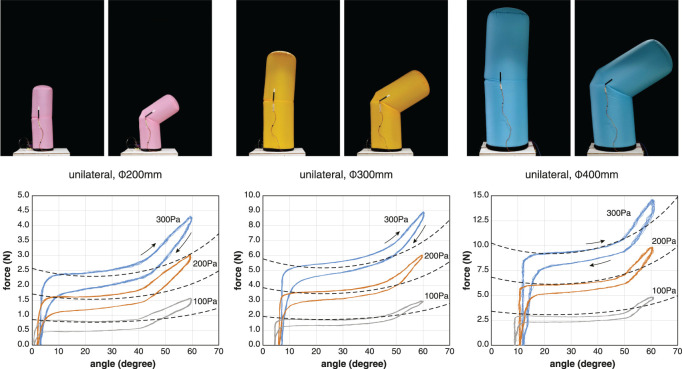
Relationships between the tensile force and angle of unilateral joints under constant pressure conditions. The dotted line represents the theoretical model. The inflatables used for the measurement were U200, U300, and U400. Plots of 10 cycles at the pressure conditions of 100 Pa, 200 Pa, and 300 Pa. The pulling angle of the tendon is 30°. Note that the scale of the vertical axis is different.

We measured the basic properties of three different sizes of unilateral inflatable joints (Figure 5). For a joint of one size, we conducted measurements at three different internal pressure conditions: 100 Pa, 200 Pa, and 300 Pa. The inflatable samples used were the U200, U300, and U400 listed in Table 1. The pulling angle of the tendon was fixed at 30° in all conditions. The designed range of motion of these inflatable joints was approximately 45°, but in practice they could be bent beyond this range. However, exceeding 60° could cause the body below the joint to buckle, so the test range was set at 0°–60°.

The results show that the tendon tension required for flexion is approximately proportional to the internal pressure and to the square of the radius. This is consistent with the theoretical model. Even in the neutral state with the tendon relaxed, a bias angle was observed that tended to increase with increasing internal pressure. This bias angle is unique to unilateral inflatable joints and results from fact that the leaf-shaped patch attached to only one side opens slightly due to internal pressure.

The tension-angle relationship shows consistent hysteresis, with the tension required for the operation being less in extension than in flexion. The hysteresis increased with larger internal pressure. Inflatable joints do not have areas where the fabric slides against each other with friction. When the joint flexes, the fabric pulled by the tendon is bent nearly 180° to form a pocket (Figure 2B). The fold line reaches almost half of the circumference of the cylindrical body and increases in length with flexion. Fibers do not exhibit an ideal elastic deformation, especially when the fold is sharp and the curvature is large, and they consume energy. We assume that the hysteresis is caused by energy consumption at the sharp angle bends when the fabric is pulled inward.

**FIGURE 6:**
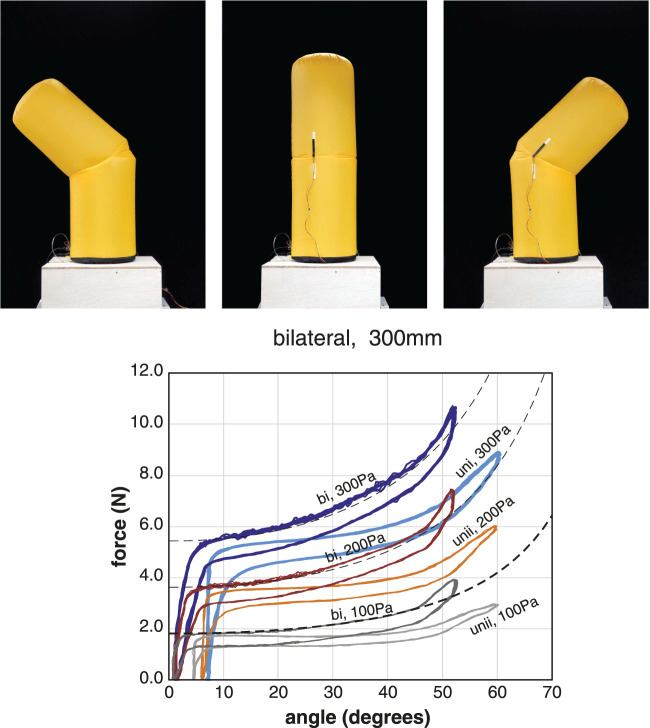
Relationships between the tensile force and angle of bilateral joints under constant pressure conditions. The dotted line represents the theoretical model of the bilateral joint. The data of the unilateral joints are also included for comparison. The inflatables used for the measurement were the B300 and U300. Plots of 10 cycles at the pressure condition of 100 Pa, 200 Pa, and 300 Pa. The pulling angle of the tendon is 30°.

We compared the basic properties of unilateral and bilateral joints (Figure 6). The advantage of the bilateral joints is that the range of motion is twice that of the unilateral joints, and there is no angular offset in the neutral state. Other differences in their properties exist. These differences result from the structure of the connection between the membrane and the tendon. In the case of the bilateral joints, the tension is transmitted to the membrane via the leaf-shaped patch; therefore, so the moment arm is small and the phase advances, as shown in Eq. 11.

We also fabricated a sample U300C with offset *α* = *π*/2, enabling the rotation axis to pass through the center of the cylindrical body. When this sample was pressurized, the force *τ* required to keep the joint straight was small, and the sample bent only by the weight of the upper body without tendon tension. As suggested by the theoretical model, the results show that the spring force of the joint can be designed by adjusting the parameter *α*.

##### 3.3.2 Results: Tendon Angle

**FIGURE 7:**
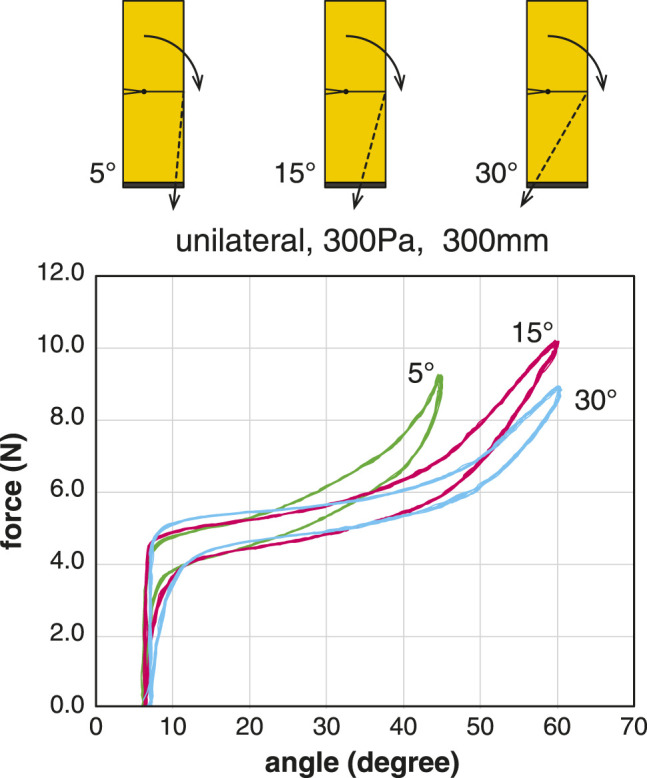
Comparison of the tension-angle relationship at different tendon angles. The inflatable used for the measurement was the U300. Plots of 10 cycles at the pressure condition of 300 Pa.

We measured the difference in the properties depending on the angle of the tendon (Figure 7). There were three different tendon angles: 5°, 15°, and 30°. Using the same sample U300, the angle of the tendon was changed by adjusting the posture of the test stand.

The relationship between the tension of the tendon and the joint angle shows that the greater the angle of the tendon from the vertical axis, the greater the range of motion can be achieved with a smaller tension force. When the tendon angle was 5°, a relatively large amount of tension was required for flexion, and when the joint angle exceeded 45°, the body below the joint began to buckle.

From Eq. 10, the moment arm *d* is at its maximum when *ϕ* follows the changing *θ* and satisfies the condition *ϕ* = *θ*. However, it is practical to set *ϕ* to a constant initial value of *ϕ*_0_. The value of *ϕ*_0_ should be half of the range of the joint angle. This explains mechanical advantage in using the pull angle of 30°.

### 3.3.3 Range of Motion

We measured and compared the basic properties of three joints with different rated ranges of motion. The inflatable samples used were U300, U300W, and U300D. These samples had leaf-shaped patches of different shapes. The sample U300W (wide) has a wider patch than sample U300 (normal). The sample U300D (double) had a patch that was a stack of two patches of the same shape as sample U300 (normal). The common measurement conditions were a pulling angle of 30° and an internal pressure of 300 Pa.

**FIGURE 8:**
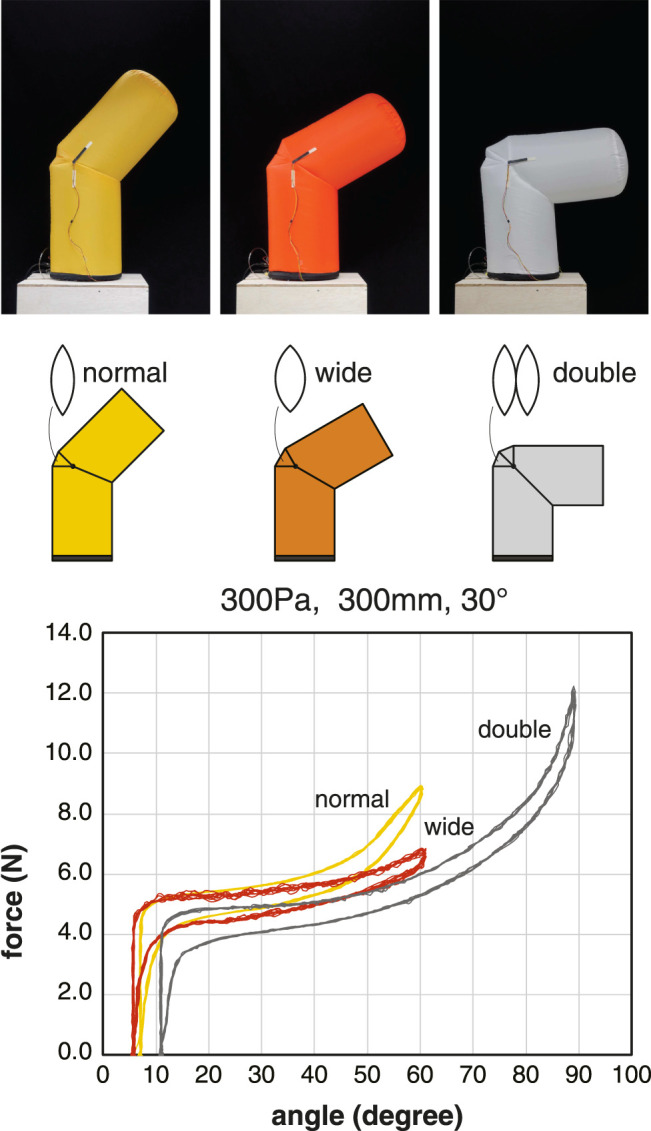
Comparison of the tension-angle relationship for different types of leaf-shaped patches. The inflatables used for the measurement were U300, U300W, and U300D. Plots of 10 cycles at the pressure condition of 300 Pa.

Based on the measurement results (Figure 8), the wider the deployment width of the leaf-shaped patch, the larger the range of motion without excessive tension. In sample U300D, a range of motion up to 90° was achieved. When the tension was increased further, the body below the joint buckled. In the U300D with double patches, a relatively large offset angle was found even in the neutral position with zero tendon tension. In addition, the two patches tried spreading out and pushing against each other. Therefore, for moving angles less than 60°, it is recommended to use a wide patch design instead of a double patch design.

### 3.3.4 Multiple Joints

The basic characteristics were measured on a single joint sample. For applications, multiple proposed joints were placed on the inflatable body. We could combine multiple joints to make a multi-degree-of-freedom joint or a serial link robot arm. Similar to the structure of the human finger, flexion of multiple joints connected in series with a single tendon could easily be achieved. When multiple joints are connected in series, the changing angle of the tendon with the flexion of the joint must be taken into account, and the path of the tendon must be carefully designed. The theoretical model of a single joint can be used if multiple joints are designed so that the parameter *ϕ* is monitored or is constant. Since the single-joint model does not include the effect of gravity, the weight of the links needs to be considered, especially in multiple joints.

**FIGURE 9:**
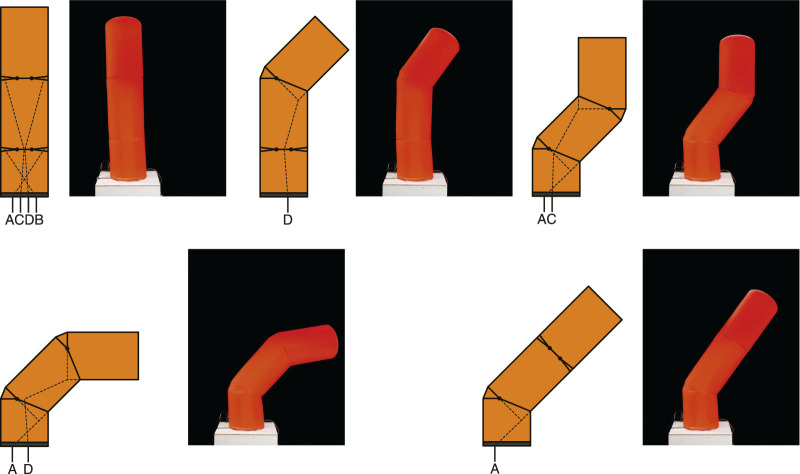
Tendon placement and various postures in an inflatable with two bilateral joints connected in series. The mirrored postures are also possible. The tendons that drive the upper joint run through the center of the lower joint.

Figure 9 shows a simple arm with two bilateral joints connected in series. A small plate with a hole is suspended inside the inflatable so that the tendon passes through the center of the lower joint. Since the upper and lower joints can be controlled independently by the tendons, the robot arm is capable of adopting a variety of postures.

### 3.4 Comparison of the Theoretical Model and Measurement Results

**FIGURE 10:**
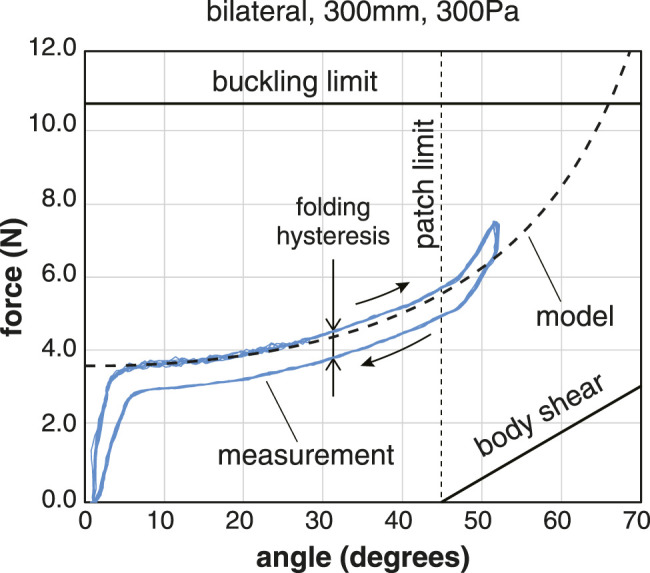
Comparison of the theoretical pressure of model and measurement results. The conditions are a bilateral joint with a diameter of 300 mm and pressure of 300 Pa. The pulling angle of the tendon is 30°. Besides the torque due to internal pressure, hysteresis related to the folding of the fabric, the gravity, the shear deformation, and the buckling limit is also involved.

The validity of the theoretical model was examined by comparing the measurement results of the inflatable joints with the predictions. The results show that the theoretical model for the torque derived from the volume change associated with flexion can predict the tension-angle relationship accurately. On the other hand, several other phenomena need to be taken into account to explain the measurement results (Figure 10).

The joint characteristics change before and after reaching the rated range of motion, which is determined by the height of the leaf-shaped patch. After the patch is fully deployed, the tension of the tendon is also spent in deforming the body itself, and the deformation of the inflatable body seems to have a spring-like property. The range of motion is also constrained by the buckling limit. Buckling is a condition in which the membrane of an inflatable loses its pre-tension due to the internal pressure, causing it to wrinkle. We observed that wrinkling occured on one side of the cylindrical body. The tension of the tendon at the buckling limit can be roughly estimated as half of the axial force that a cylindrical inflatable can withstand, as follows.

$$F_{buckling}=\frac{\pi r^{2}P}{2}$$ (16)

A continuos pulling of the tendon beyond the rated angle causes shear deformation of the lower body, resulting in an elastic force. This force can be estimated approximately as follows.

$$F_{shear}=\pi r^{2}P\sin\left(\frac{r\theta }{L}\right)$$ (17)

## 4 Physical Human-Robot Interaction

### 4.1 Hug Robot

**FIGURE 11:**
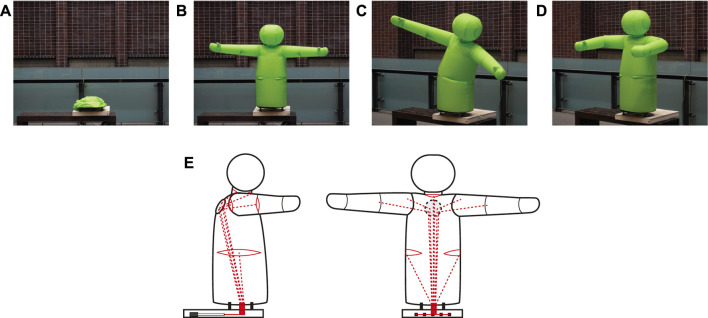
Inflatable hug robot. **(A)**: deflated state, **(B)**: inflated state, **(C)**: tileted state with the trunk joint, **(D)**: hugging posture with bent shoulders and elbows, and **(E)**: Schematic diagram showing wire routing.

We developed a prototype robot for physical human-robot interactions (pHRI) that combining multiple proposed joint designs (Figure 11). Non-verbal communication through physical contact was included in pHRI. To demonstrate the unique features of inflatable robots, we focused on a hugging robot that having a similar shape as that of the upper human body.

The robot has unilateral inflatable joints in the shoulders and elbows, and bilateral joints in the neck and waist. The unilateral joints require one tendon per joint for the operation, while the bilateral joints require two tendons per joint. Shoulders are capable of horizontal adduction and elbows are capable of flexion (Figure 11D). The neck joint is capable of nodding and performing upward movements. The trunk joint can tilt the upper body to the left or right (Figure 11C). The wires leading to the joints other than the trunk joint are passed through the pulley plate on the back of the robot to obtain the suitable pulling angle (Figure 11E). All the tendons are pulled into a thin box at the base. The group of linear actuators that pull the tendons is enclosed in the box, unlike the test stand used to measure the basic characteristics. The shoulder and arm joints are moved synchronously on both sides, and the neck is driven only by nodding, resulting in the reduction of the number of linear actuators to five. The linear actuator used has a stroke of 200 mm and a gear ratio of 22:1 (P16 series, Actuonix Motion Devices Inc.). Because a large stroke is required to drive the elbow joint through the shoulder joint, a dynamic pulley is used to double the stroke.

### 4.2 Results

Detecting physical contact is the basis of pHRI. Although there are no sensors mounted on the surface of the hug robot, a single pressure sensor is installed for feedback control of the internal pressure by the blower. We investigated the changes in the internal air pressure during contacts. Figure 13 shows the change in the internal air pressure in four different touch patterns: strong hug, light hug, thump, and handshake. The results showed that even during active stabilization of the internal pressure, human contact was noticeable as a change in the internal pressure. The internal pressure increased upon contact, decreased upon leaving, and then converged to the target pressure. Large or quick volume changes appeared as large amplitude changes in internal pressure. On the other hand, in a tapping contact, the pressure change is difficult to detect via the amplitude alone. Future experiments can verify whether these contacts can be identified by pattern recognition of the waveforms. Moreover, since the position of the contact is essential information for interactions, we could consider detecting the contact area by adding sensors.

**FIGURE 12:**
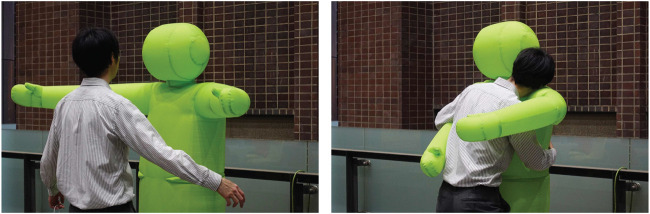
Inflatable hug robot.

**FIGURE 13:**
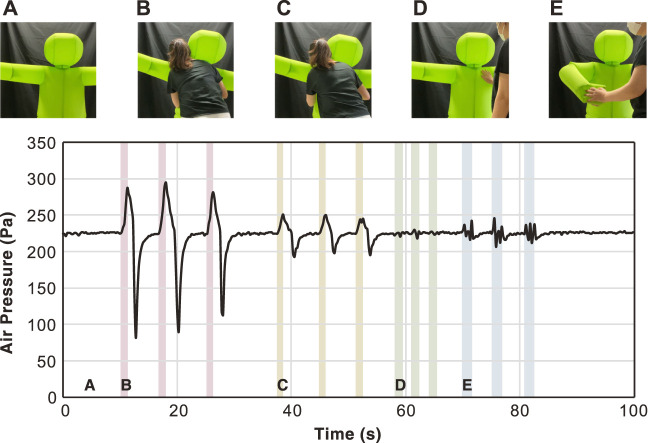
Changes in air pressure caused by physical interactions. **(A)**: Neutral, **(B)**: Hugging tightly, **(C)**: Light hug, **(D)**: Thumping, and **(E)**: Handshake

## 5 Conclusion

We have proposed a soft inflatable joints driven by inner tendons. Because the inner pressure was continuously controlled by an electric blower, a small air leak or opening and closing of the inflatable is acceptable. The experiments with different design parameters revealed differences in basic properties depending on the size, internal pressure, and pulling angle. We have also developed a theoretical model of the inflatable joint to design and estimate the properties of the joints. The theoretical model predictions matched the measurement results. In addition to the evaluation of single joints, we explored the serial connection of multiple joints and the design of a humanoid robot. The proposed joint mechanism contributes to the design of soft articulated robots. Further HRI experiments by integrating sensors and display devices into the inflatable robot will be performed in our future study.

## Data Availability

The raw data supporting the conclusions of this article will be made available by the authors, without undue reservation.
